# Diagnostic Accuracy of Mucosal Biopsy versus Endoscopic Mucosal Resection in Barrett's Esophagus and Related Superficial Lesions

**DOI:** 10.1155/2015/735807

**Published:** 2015-01-29

**Authors:** Hany M. Elsadek, Mamdouh M. Radwan

**Affiliations:** ^1^Gastroenterology Unit, Internal Medicine Department, Faculty of Medicine, Zagazig University, Zagazig, Egypt; ^2^Pathology Department, Royal Commission Medical Center (RCMC), Yanbu, Saudi Arabia

## Abstract

*Background*. Endoscopic surveillance for early detection of dysplastic or neoplastic changes in patients with Barrett's esophagus (BE) depends usually on biopsy. The diagnostic and therapeutic role of endoscopic mucosal resection (EMR) in BE is rapidly growing. *Objective*. The aim of this study was to check the accuracy of biopsy for precise histopathologic diagnosis of dysplasia and neoplasia, compared to EMR in patients having BE and related superficial esophageal lesions. *Methods*. A total of 48 patients with previously diagnosed BE (36 men, 12 women, mean age 49.75 ± 13.3 years) underwent routine surveillance endoscopic examination. Biopsies were taken from superficial lesions, if present, and otherwise from BE segments. Then, EMR was performed within three weeks. *Results*. Biopsy based histopathologic diagnoses were nondysplastic BE (NDBE), 22 cases; low-grade dysplasia (LGD), 14 cases; high-grade dysplasia (HGD), 8 cases; intramucosal carcinoma (IMC), two cases; and invasive adenocarcinoma (IAC), two cases. EMR based diagnosis differed from biopsy based diagnosis (either upgrading or downgrading) in 20 cases (41.67%), (Kappa = 0.43, 95% CI: 0.170–0.69). *Conclusions*. Biopsy is not a satisfactory method for accurate diagnosis of dysplastic or neoplastic changes in BE patients with or without suspicious superficial lesions. EMR should therefore be the preferred diagnostic method in such patients.

## 1. Introduction

Barrett's esophagus (BE) is a sequel of gastroesophageal reflux disease (GERD). Prevalence of BE in western countries is about 2% in general population and around 5–15% in chronic GERD patients [1, 2]. BE is a premalignant lesion that may progresses through stages of dysplasia to cancer, with esophageal adenocarcinoma (EAC) occurring at an overall incidence rate of 0.4–0.5% per year [3]. The incidence of EAC in BE cases with high-grade dysplasia (HGD) is above 6% [4]. There are worldwide different endoscopic surveillance protocols for patients with BE and different grades of dysplasia; however, the commonest surveillance frequency used is every 3–5 years for BE without dysplasia, every 6–12 months for BE with LGD (low-grade dysplasia), and every 3 months for BE with HGD without intervention [5–8]. There is a lack of agreement concerning the optimal management of dysplasia and early EAC. There remains heterogeneity in the management of HGD/early EAC throughout the world; the primary options include managing HGD with surveillance alone, endoscopic therapy, or surgical resection (esophagectomy) for HGD and early EAC [6–8].

The main role of EMR in BE patients is the curative treatment of prominent lesions and neoplasms without lymph node involvement or distant metastases. Thus, its use in correct indication requires a correct disease staging which can include endoscopic, histologic, and sometimes radiographic criteria [9]. Endoscopic mucosal resection is used for the en bloc excision of lesions smaller than 2 cm or for the resection of greater lesions in various fragments, which is called a “piecemeal” resection [9]. Technically, EMR entails several systematic steps of which submucosal injection is very useful; it allows the creation of a “security chamber” that minimizes the complication risks. Subsequent resection techniques can then be performed [10–13].

Upon endoscopic surveillance, four quadratic biopsies are to be taken from BE segment. Visible prominent lesions found related to BE segment are indicated for biopsy sampling or even endoscopic resection. Further endoscopic interventions or surgical interference may be indicated based on the results of histopathologic examination, such as radiofrequency ablation (RFA) for HGD or IMC (intramucosal carcinoma) and esophagectomy for invasive adenocarcinoma (IAC) [7]. Recent publications have reported the development of EAC in patients who were treated by RFA for HGD in BE field [14, 15]. These reports should raise the speculation that these patients possibly had cancer from the start, but not detected by standard biopsy. On the basis of this report, the decision to carry out this prospective study was made. The aim of this prospective study was to clear whether the agreement in histopathologic diagnosis between forceps biopsy and EMR samples is sufficient when examining BE and its related superficial lesions.

## 2. Methods

This prospective study was done at gastroenterology and pathology departments, Royal Commission Medical Center (RCMC), Yanbu, Saudi Arabia, during the period from June 2011 to June 2014. The study was performed on 48 patients undergoing programmed upper gastrointestinal endoscopy for surveillance of previously diagnosed BE, either without dysplasia (34 patients) or with dysplasia (14 patients). Patients were categorized into two groups. Group A comprised 24 patients in whom grossly apparent superficial lesions suspicious of neoplasia were found in relation to the BE segment; these were type 0 lesions according to Paris classification of suspected neoplastic lesions in the digestive tract (Table 1) [16]. Group B comprised 24 patients with no grossly apparent suspicious lesions in relation to the BE segment. Exclusion criteria in this study included a previous endoscopic interventional therapy, advanced EAC with lymph node or distant metastasis (as the diagnosis was no more questionable), and esophageal lesions that were anatomically not related to the BE segment or morphologically exceeding the type 0 (superficial) lesion described in Paris classification, as well as any interobserver variation encountered during pathologic interpretation. Moreover, patients who had uncorrected coagulopathy or any contraindication to standard endoscopy such as severe cardiopulmonary comorbidities were excluded.

All patients received information concerning the techniques used and their possible complications. Informed consent for participation in the study was obtained from every patient. An approval from the hospital (RCMC) ethics committee was also obtained before proceeding to the study.

All endoscopies were performed by an experienced endoscopist, as outpatient procedures and under deep sedation controlled by an anesthesiologist. Upper gastrointestinal endoscopy was done using high definition endoscopy and narrow band imaging (NBI) (GIF H180, Olympus). After the introduction of endoscope, esophageal mucosa was rinsed with water, and BE segment and its related superficial lesions were delineated. For lesions with poorly defined margins chromoendoscopy with indigo carmine stain was used.

A detailed view (using magnification and NBI) of the BE segment was recorded and four quadratic biopsies were taken. The endoscopic appearance of type 0 lesions was assessed and described according to Paris classification (Table 2), and targeted biopsies were taken from them.

Endoscopic resection was performed in another endoscopic session (as it was technically difficult to do in the same session with biopsy), but not more than three weeks later to avoid false positive results of EMR examination that may result from fibrosis at the biopsy site [17]. EMR was performed in all patients (both groups A and B). In group B, EMR samples were taken from BE segments, even without previous known history of dysplasia, for study purposes. Lesions were marked circumferentially using argon plasma with a 40 W power. Submucosal injection was then performed with isotonic saline. Techniques used for mucosal resection after submucosal injection were loop resection, cap-assisted resection, and band ligator-assisted resection [10–13, 18]. Surgical backup was available for the event of uncontrolled hemorrhage or perforation.

### 2.1. Histopathologic Processing

In accordance with a previously published protocol [19], all EMR specimens were marked with India ink along their lateral and deep margins, then were stretched and pinned to wax blocks, fixed in 10% formaldehyde for 24 hours, and then serially sectioned at 2 mm intervals before routine histologic processing of all tissue. Sections were stained with H&E for microscopic analysis.

The histologic diagnosis and grading of every case were done by one expert pathologist and confirmed by a second opinion of another pathologist. Classification of the lesions on histopathologic examination was based on previously published criteria [20], in accordance with the Vienna classification of gastrointestinal epithelial neoplasia [21]. IAC was diagnosed when malignant cells, singly or in groups, infiltrate beyond the basement membrane. Staging of the lesions was completed by CT scan and/or endoscopic ultrasonography if necessary.

### 2.2. Statistical Evaluation

Basic methods of descriptive statistics were used, for example, mean and standard deviation. Interrater agreement between biopsy based diagnoses and EMR based diagnoses was determined by using the kappa statistic. The strength of rater agreement was categorized as follows: 0.00–0.20: slight; 0.21–0.40: fair; 0.41–0.60: moderate; 0.61–0.80: substantial; 0.81–1.00: almost perfect. Corresponding 95% confidence interval (CI) for the kappa value was calculated [22].

## 3. Results

**Figure 1:**
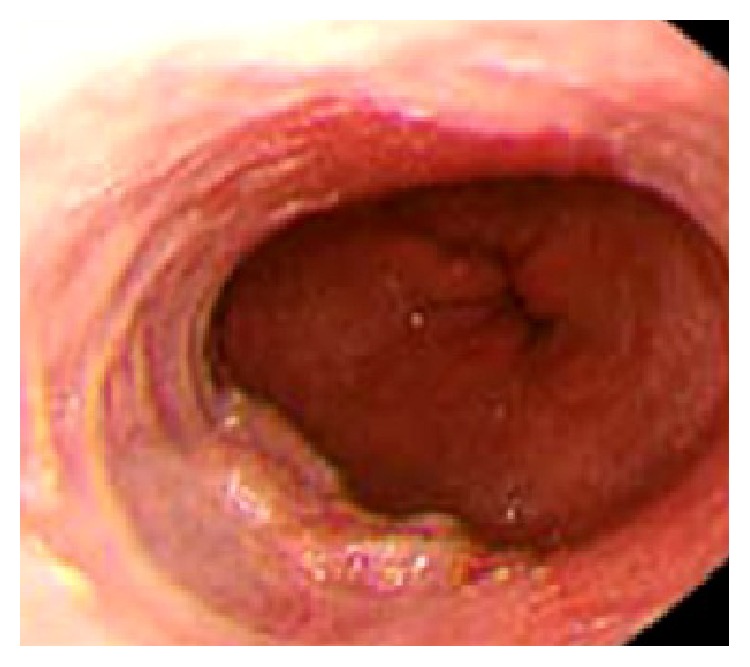
Superficial type 0-Is lesion.

Overall, we studied 48 BE patients (36 men, 12 women, mean age 49.75 ± 13.3 years, range 32–83 years). In group A (*n* = 24), superficial lesions related to the BE segment were found that were according to Paris classification, type 0-Is (four cases) (Figure 1), type 0-IIa (12 cases), type 0-IIb (six cases), and type 0-IIc (two cases). Forceps biopsies and EMR specimens were taken from these lesions. In group B (*n* = 24), no obvious lesions related to BE segment were found and, hence, biopsies and EMR specimens were taken from BE segments.

**Figure 2:**
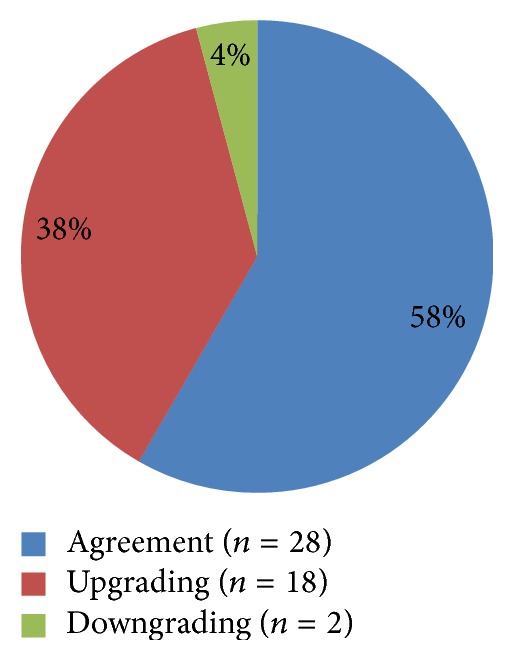
Agreement and disagreement between biopsy based and EMR based diagnoses.

The differences in histopathologic diagnoses among all patients according to the type of the specimen (biopsy versus EMR) were shown in Table 3. According to kappa statistic of interrater agreement, the agreement and disagreement between biopsy based and EMR based histopathologic diagnoses were described in Figure 2 and Table 3. Agreement between biopsies and EMR was found only in 28 cases (58.33%), while disagreement between them was found in 20 cases (41.67%) (Kappa = 0.430, 95% CI: 0.170–0.690) and the strength of agreement is considered to be “moderate.” An “upgrading” diagnosis was made by EMR (i.e., a higher degree of dysplasia or neoplasia than that diagnosed with biopsy) in 18 cases (37.5%), and a “downgrading” diagnosis was made by EMR (i.e., a lower degree of dysplasia or neoplasia than that diagnosed with biopsy) in two cases (4.17%).

The biggest disagreement between biopsy based and EMR based diagnoses was found in cases diagnosed as LGD with biopsy (28 patients). Out of these 28 patients, 16 cases (57.14%) showed different EMR based diagnoses, with upgrading to HGD in four of them and to IMC in four of them. The second big disagreement was found in those with biopsy finding of HGD (eight patients); of them, four patients (50%) showed different EMR based diagnoses, with upgrading to IMC in two cases and downgrading to NDBE in two cases (Table 3). In this study, there were only two cases of IAC (without any lymph node or distant metastasis) that were diagnosed in agreement between biopsy and EMR (Table 3).

There was no significant difference between group A patients and group B patients regarding the degree of agreement between biopsy based and EMR based diagnoses (*P* = 1), as there were 14 cases of agreement and 10 cases of disagreement in each group (Table 4).

Mild to moderate bleeding was seen at most EMR sites that stopped immediately spontaneously. Only in two cases, bleeding persisted after EMR and was managed successfully by the application of bipolar coagulation. Apart from this bleeding, no other complications were encountered in this study.

## 4. Discussion

The importance of endoscopic surveillance of BE for early detection of dysplastic or neoplastic changes is well established [6, 23]. Histopathologic examination of the mucosal sample enables not only an accurate diagnosis but also grading of dysplasia and neoplasia.

During endoscopic surveillance of BE, biopsies are usually taken from BE segment, and either biopsy or endoscopic resection is used for visible prominent lesions found related to BE segment. Subsequently, further endoscopic interventions or surgical interference may be indicated based on histopathologic results [7].

The question was whether biopsies from BE segment or its related esophageal lesions are sufficient for accurate diagnosis and histologic grading. The aim of this prospective study was to clear whether the agreement in histopathologic diagnosis between forceps biopsies and EMR is sufficient when examining BE and its related superficial lesions.

In this study, done on 48 patients with BE undergoing surveillance endoscopy, an agreement between biopsy based and EMR based diagnoses was found only in 28 cases (58.33%), and a disagreement between them was found in 20 cases (41.67%). An EMR based upgrading diagnosis was made in 18 cases (37.5%), while an EMR based downgrading diagnosis was made by EMR in two cases (4.17%). The rates of agreement and disagreement between biopsy and EMR were identical among patients with sampled mucosa from BE (group B) or from related esophageal superficial lesions (group A). The biggest disagreement (57.14%) between biopsy and EMR diagnoses was found in patients with biopsy finding of LGD; the second big disagreement (50%) was found in patients with biopsy finding of HGD.

Multiple studies showed results consistent with the findings of this study. Larghi et al. found in a series of 40 BE patients undergoing EMR that six of 25 (24%) patients diagnosed initially with HGD were upgraded to IMC and six of 15 (40%) patients with IMC were upgraded to invasive EAC [24]. In the single-center study of Chennat et al., including 49 BE patients, EMR resulted in a change of diagnosis in 22 (44.8%) patients (upstaging for 14% and downstaging for 31%) compared with pre-EMR biopsy results [19]. In their study on the effect of EMR on histologic grading and staging for 75 BE patients with biopsy-proved HGD or EAC, Moss et al. reported that EMR resulted in a change of diagnosis for 48% of patients (downstaging for 28% and upstaging for 20%) [25].

Similar results to the findings of the current study were concluded from a recent multicenter study of Wani et al., done on 138 known patients of BE (with or without endoscopically visible lesions) undergoing endoscopic eradication therapy; EMR resulted in a change of diagnosis for 31.1% patients (upgrade 10.1% and downgrade 21%) [26]. The discrepancy between the two studies regarding the frequency of EMR based upgrading/downgrading could be explained by the different inclusion criteria, as only selected histologic grades of BE lesions were included in the study of Wani et al., while all histologic grades were included in the current prospective study.

Multiple retrospective studies show that examination of the EMR samples brings greater interobserver agreement (among pathologists) of BE and its related neoplasia compared to biopsied samples, and these studies have suggested that the diagnostic yield of EMR is higher compared to biopsies [27–29].

There were only two cases of IAC in this study that were diagnosed in agreement between biopsy and EMR. Both patients with IAC were referred for surgery. Moreover, 18 patients with established EMR based diagnosis of HGD (10 cases) or IMC (eight cases) (Table 3) were referred for endoscopic intervention (RFA). Out of these 18 cases referred for RFA, 10 cases (55.56%) had previous biopsy based lower grade diagnoses (NDBE/LGD) that would not mandate any intervention. From above findings, more than half of the cases indicating endoscopic intervention could miss the needed intervention if the diagnosis was based only on biopsy result.

Consistent with the above results, a group of pathologists from Japan and Germany have recently reported that, with regard to BE related early neoplasia (HGD/IMC), the indications for endoscopic intervention or major surgery cannot be decided on the basis of biopsy histology, and the choice between them should be made according to the invasion depth known after mucosal resection [30]. They added that such lesions should not be managed by endoscopic ablation (e.g., RFA) alone but by endoscopic resection (EMR/submucosal dissection) because components of invasive carcinoma are frequently present in the mucosa and submucosa and knowledge obtained from resected mucosal samples is needed for additional therapy [30].

The combination of EMR/RFA is the gold standard for the treatment of early neoplasms of the esophagus at the field of BE. This treatment leads to the eradication of neoplasia in 90% of patients and the percentage of recurrence is almost nil [31]. Ablation therapy (RFA) is, however, exceptionally indicated solely, in BE patients with HGD with long segments of flat-type mucosa (without visible lesion), where endoscopic resection is burdened with complications, especially the emergence of stenosis [32].

An alarming report has been published by American authors regarding the detection of adenocarcinoma in three patients who underwent RFA for HGD in the field of BE. Carcinoma was diagnosed few months after the end of RFA treatment [14]. It can be assumed that carcinoma could have been there, when RFA was performed, but could not be caught up by simple biopsy based histopathologic examination. Although in-depth discussion of that finding is beyond the scope of the current study, reason to believe that cancer was present at the time of RFA treatment is the presence of carcinoma in the surgical resection specimens from a significant percentage (sometimes around one-third) of patients with an initial diagnosis of HGD, who had been in the past decade indicated for esophagectomy [33].

The discrepancy between the EMR and biopsy may be due to several reasons. The first is undoubtedly good size and orientation of the sample after endoscopic resection, as well as the ability to evaluate mucosal landmarks, such as double muscularis mucosae. Another reason for this difference is that the sample of EMR includes (in most cases) a part of the submucosa and hence is better evaluated [26, 28].

Finally, we concluded that standard biopsies are not sufficient for accurate diagnosis and classification of dysplasia and neoplasia in the esophagus in patients with BE and its related superficial esophageal lesions. EMR is crucial before proceeding to endoscopic ablation therapy or surgical interference in such patients.

## Figures and Tables

**Table 1:** Macroscopic classification of digestive tract neoplasms.

Superficial type 0	Superficial protruding or nonprotruding lesions

Advanced type 1	Protruding carcinoma, attached on a wide base

Advanced type 2	Ulcerated carcinoma with sharp and raised margins

Advanced type 3	Ulcerated carcinoma without definite limits

Advanced type 4	Nonulcerated, diffusely infiltrating carcinoma

Advanced type 5	Unclassifiable advanced carcinoma

**Table 2:** Macroscopic classification of type 0 lesions.

Protruding	
Pedunculated	0-Ip
Sessile	0-Is
Nonprotruding and nonexcavated	
Slightly elevated	0-IIa
Completely flat	0-IIb
Slightly depressed	0-IIc
Elevated and depressed types	0-IIc + IIa or 0-IIa + IIc
Excavated	
Ulcer	0-III
Excavated and depressed types	0-IIc + III or 0-III + IIc

**Table 3:** Agreement and disagreement in histopathologic diagnosis between biopsies and EMR; *the agreement is marked with underline and bold numbers and the upgrading cases lie on the right side to agreement cases, while downgrading cases lie on the left side to agreement cases*.

			EMR based histologic diagnosis															
			NDBE *n* = 16			LGD *n* = 12			HGD *n* = 10			IMC *n* = 8			IAC *n* = 2			Kappa statistic
			gp A	gp B	T	gp A	gp B	T	gp A	gp B	T	gp A	gp B	T	gp A	gp B	T	
Biopsy based histologic diagnosis	NDBE	*n* = 22	2	12	**14**	2	4	6	0	2	2	0	0	0	0	0	0	**Kappa** = 0.430 **95% CI**: 0.170–0.690
	LGD	*n* = 14	0	0	0	4	2	**6**	0	4	4	4	0	4	0	0	0	
	HGD	*n* = 8	1	0	2	0	0	0	4	0	**4**	1	0	2	0	0	0	
	IMC	*n* = 2	0	0	0	0	0	0	0	0	0	2	0	**2**	0	0	0	
	IAC	*n* = 2	0	0	0	0	0	0	0	0	0	0	0	0	2	0	**2**	

gp A = group A, gp B = group B, and T = total.

**Table 4:** Comparison between group A patients and group B patients regarding agreement between biopsy based and EMR based diagnoses.

	Group A		Group B		Total		*P* value
	Number	%	Number	%	Number	%	
Agreement	**14**	**29.17**	**14**	**29.17 **	**28**	**58.33**	**1.00**
Disagreement	**10**	**20.83**	**10**	**20.83**	**20**	**41.67**	
Upgrading	8	16.67	10	20.83	18	37.50	
Downgrading	2	4.17	0	0.00	2	4.17	
Total	**24**	**50.00**	**24**	**50.00**	**48**	**100.00**	
